# Data for modeling nitrogen dioxide concentration levels across Germany

**DOI:** 10.1016/j.dib.2021.107324

**Published:** 2021-08-26

**Authors:** Markus Fritsch, Svenia Behm

**Affiliations:** Chair of Statistics and Data Analytics, University of Passau, Germany

**Keywords:** Air pollution, Nitrogen dioxide, Corine land cover, EEA air quality data

## Abstract

The described secondary data provide a comprehensive basis for modeling conditional mean nitrogen dioxide (NO_2_) concentration levels across Germany. Besides concentration levels, meta data on monitoring sites from the German air quality monitoring network, geocoordinates, altitudes, and data on land use and road lengths for different types of roads are provided. The data are based on a grid of resolution 1 × 1 km, which is also included. The underlying raw data are open access and were retrieved from different sources. The statistical software R was used for (pre-)processing the data and all codes are provided in an online repository. The data were employed for modeling mean annual NO_2_ concentration levels in the paper “Agglomeration and infrastructure effects in land use regression models for air pollution – Specification, estimation, and interpretations” by Fritsch and Behm (2021).

## Specifications Table

**Table:** 

Subject	Environmental Science
Specific subject area	Pollution
Type of data	Table
How data were acquired	Open access datasets from different sources were retrieved, (pre-)processed, and combined in the statistical software R[2].
Data format	Analyzed Filtered
Parameters for data collection	Mean annual nitrogen dioxide (NO_2_) concentration levels observed at the sites of the German air quality monitoring network in 2015; corresponding meta data on monitoring sites, geocoordinates, and altitudes; data on population density and administrative regions, land use via land cover classes, and road traffic network via lengths of different types of roads; data were obtained based on a 1 × 1 km grid of Germany, which is also provided.
Description of data collection	Open access datasets from different sources were retrieved, (pre-)processed and combined in the statistical software R. All employed codes are provided in a repository hosted online at https://doi.org/10.5281/zenodo.5148684 ([9]).
Data source location	Germany Primary data sources:
	• Air Quality e-Reporting [3]
	• CORINE land cover data [4]
	• German boundary [5]
	• Administrative regions at municipality level [6]
	• Road traffic network [7]
	• Digital terrain model grid [8]
Data accessibility	The secondary data described in this paper can be downloaded from a repository hosted online at https://doi.org/10.5281/zenodo.5148684 ([9]).
Related research article	M. Fritsch, S. Behm, Agglomeration and infrastructure effects in land use regression models for air pollution – Specification, estimation, and interpretations, Atmos. Environ., Vol. 253, 118337; https://doi.org/10.1016/j.atmosenv.2021.118337.

## Value of the Data

• Data are useful to investigate mean annual nitrogen dioxide (NO_2_) concentration levels, underlying spatial heterogeneities, and their relationship with population density, land use, and road traffic infrastructure.
• Researchers interested in air quality assessment, modeling of air pollutants, and corresponding validation techniques can benefit from these data.
• Data can be used by researchers to contrast different modeling techniques and validation schemes, to replicate the empirical results in [1], or for didactic purposes.
• Local air quality assessment based on background NO_2_ concentration levels can be illustrated with the data.
• Extending the data by further variables (or variables on grids of higher resolution) is straightforward; additional monitoring sites can also be added.
• Other pollutants can be investigated based on the gridded data.

## 1 Data Description

This paper describes the two secondary datasets monSitesDE and gridDE. Dataset monSitesDE contains 403 observations (rows) of 26 variables (columns). Each row of the dataset represents one site of the German air quality monitoring network provided by the European Environment Agency [3] and records the following information: Identification codes according to AirBase, annual mean NO_2_ concentration levels for 2015, geocoordinates, altitudes, monitoring site type, population density, land use indicated by different land cover classes, road lengths for different types of roads, and the German federal state in which the monitoring site is located. Dataset gridDE represents Germany as a 1 × 1 km grid. The dataset contains 356,793 grid cells (rows) and 23 variables (columns). For each grid cell, the information given in the columns refers to the grid cell centers and comprises: Grid cell identifier, geocoordinates, altitude, land use indicated by different land cover classes, municipality key, population density, road lengths for different types of roads, and the German federal state in which the grid cell is located. Table 1 summarizes all variables included in the two data sets.

**Table 1:** Overview and brief description of variables contained in datasets monSitesDE and gridDE; variables marked with * are provided in monSitesDE only, ** indicate that the variables are included in gridDE only.

Variable	Description
AQeCode*	Identification code of monitoring site according to AirBase
Y*	Mean annual NO_2_ concentration level (in $\mu {\text{g/m}}^{3}$)
Year*	Year of observation
Projection*	Coordinate reference system of geocoordinates longitude and latitude
Lon*	Geocoordinate longitude (decimal degrees) of monitoring site location
Lat*	Geocoordinate latitude (decimal degrees) of monitoring site location
AQeType*	Type of site: Background, industrial, or traffic
AQeArea*	Area surrounding site: Urban, suburban, rural, rural-nearcity,rural-regional, or rural-remote
ID**	Grid cell identifier
Lon.GK3**	Geocoordinate longitude (Gauss-Krüger) of grid cell center
Lat.GK3**	Geocoordinate latitude (Gauss-Krüger) of grid cell center
Lon.WGS84**	Geocoordinate longitude (decimal degrees) of grid cell center
Lat.WGS84**	Geocoordinate latitude (decimal degrees) of grid cell center
Alt	Altitude (meters above sea level) of monitoring site or grid cell center
HighDens	Proportion of high density residential area within buffer of radius 1km
LowDens	Proportion of low density residential area within buffer of radius 1km
Ind	Proportion of industrial area within buffer of radius 1km
Transp	Proportion of area attributed to transport within buffer of radius 1km
Seap	Proportion of area attributed to seaport within buffer of radius 1km
Airp	Proportion of area attributed to airport within buffer of radius 1km
Constr	Proportion of area attributed to construction within buffer of radius 1km
UrbGreen	Proportion of area attributed to urban green spaces within buffer of radius 1km
Agri	Proportion of agricultural area within buffer of radius 1 km
Forest	Proportion of forestry area within buffer of radius 1 km
AGS	Municipality key
PopDens	Population density (inhabitants per km^2^) at municipality key level
PriRoad	Primary roads (length in meters) within buffer of radius 1 km
SecRoad	Secondary roads (length in meters) within buffer of radius 1 km
FedAuto	Federal autobahn (length in meters) within buffer of radius 1 km
LocRoute	Local routes (length in meters) within buffer of radius 1 km
IndRegions	German federal state in which monitoring site or grid cell center is located

**Fig. 1:**
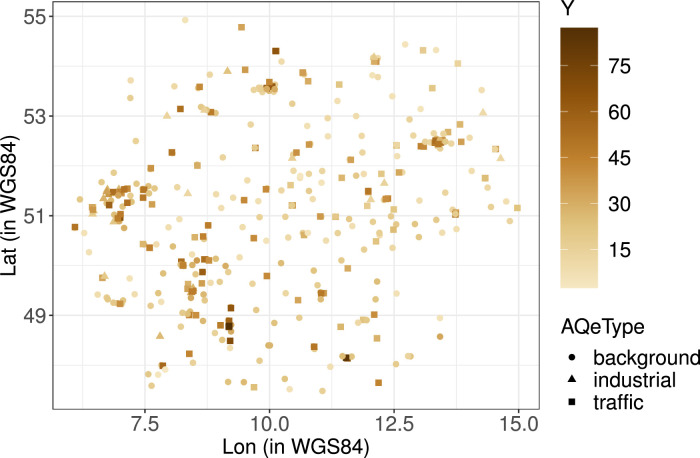
Locations of monitoring sites in dataset monSitesDE; shape of points marks type of monitoring sites (AQeType): Background (circles), industrial (triangles), and traffic (squares); coloring of the shapes represents mean annual NO_2_ concentration level (Y); darker shades of brown indicate higher levels.

Fig. 1 displays the locations of the 403 monitoring sites included in dataset monSitesDE based on the geocoordinates longitude (Lon) and latitude (Lat). The different shapes of the points mark the types of monitoring sites: Background (circles), industrial (triangles), and traffic (squares). The coloring of the shapes represents mean annual NO_2_ concentration levels at the respective monitoring sites, with darker shades of brown indicating higher levels.

Table 2 includes descriptives for each continuous variable in dataset monSitesDE – except the geocoordinates. For each variable, mean, standard deviation, minimum, lower quartile, median, upper quartile, and maximum are given.

**Table 2:** Descriptives on empirical distribution of all continuous variables in monSitesDE except geocoordinates; mean, standard deviation, and five number summary are given.

Variable	Mean	SD	Min	Q25	Median	Q75	Max
Y	25.39	14.79	2.53	14.73	22.41	33.24	87.23
Alt	197.40	215.60	0.00	45.00	112.00	297.00	1205.00
HighDens	0.04	0.10	0.00	0.00	0.00	0.01	0.78
LowDens	0.46	0.27	0.00	0.27	0.51	0.66	1.00
Ind	0.12	0.14	0.00	0.00	0.08	0.19	0.91
Transp	0.02	0.04	0.00	0.00	0.00	0.00	0.26
Seap	0.00	0.03	0.00	0.00	0.00	0.00	0.39
Airp	0.01	0.05	0.00	0.00	0.00	0.00	0.64
Constr	0.00	0.01	0.00	0.00	0.00	0.00	0.15
UrbGreen	0.05	0.08	0.00	0.00	0.00	0.09	0.42
Agri	0.15	0.24	0.00	0.00	0.01	0.20	1.00
Forest	0.11	0.24	0.00	0.00	0.00	0.07	1.00
PopDens	1194.96	1071.90	0.00	311.98	929.92	1840.84	4653.18
PriRoad	1352.82	1407.39	0.00	0.00	1222.63	2240.24	5567.02
SecRoad	331.21	711.43	0.00	0.00	0.00	0.00	3875.94
FedAuto	191.85	613.91	0.00	0.00	0.00	0.00	4321.96
LocRoute	151.59	504.19	0.00	0.00	0.00	0.00	3168.37

**Fig. 2:**
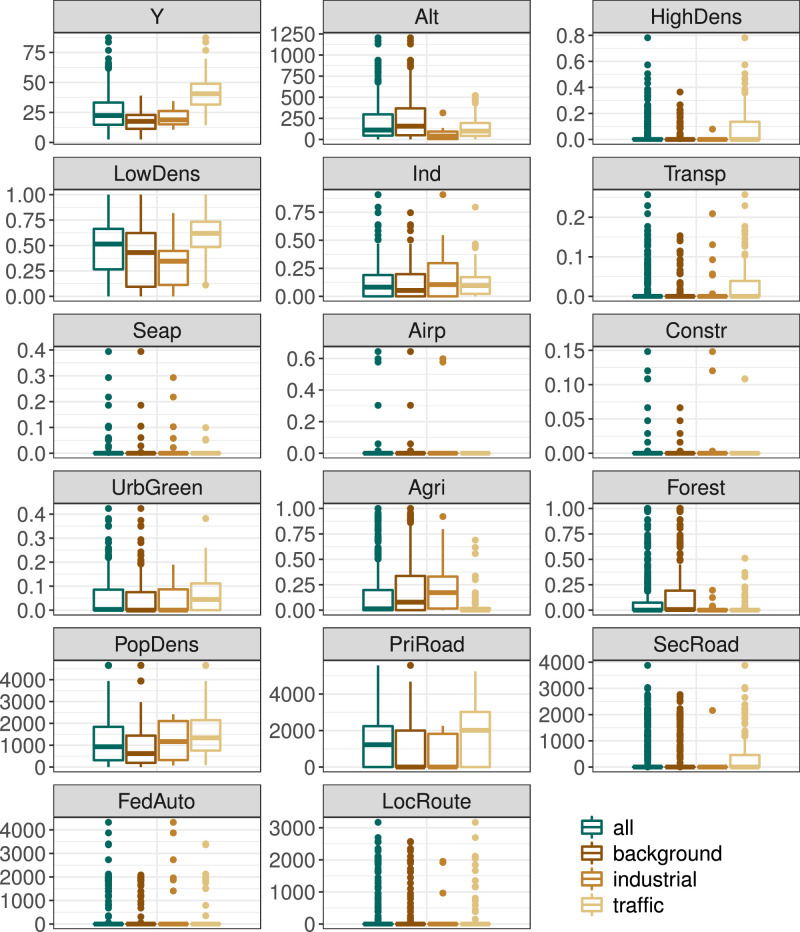
Boxplots for different monitoring site types for all continuous variables in monSitesDE except geocoordinates; color of boxplots indicates monitoring site type: dark green (all); dark brown (background); light brown (industrial); gold (traffic).

The plots provided in Fig. 2 also refer to dataset monSitesDE and show boxplots for all continuous variables – except the geocoordinates. In each of the 17 plots, four boxplots are given. The different colors of the boxplots represent the different types of monitoring sites (AQeType). The dark green colored boxplots are based on all monitoring sites in dataset monSitesDE and visualize similar information as Table 2. The three other boxplots are based on background (dark brown), industrial (light brown), and traffic (gold) monitoring sites only.

**Fig. 3:**
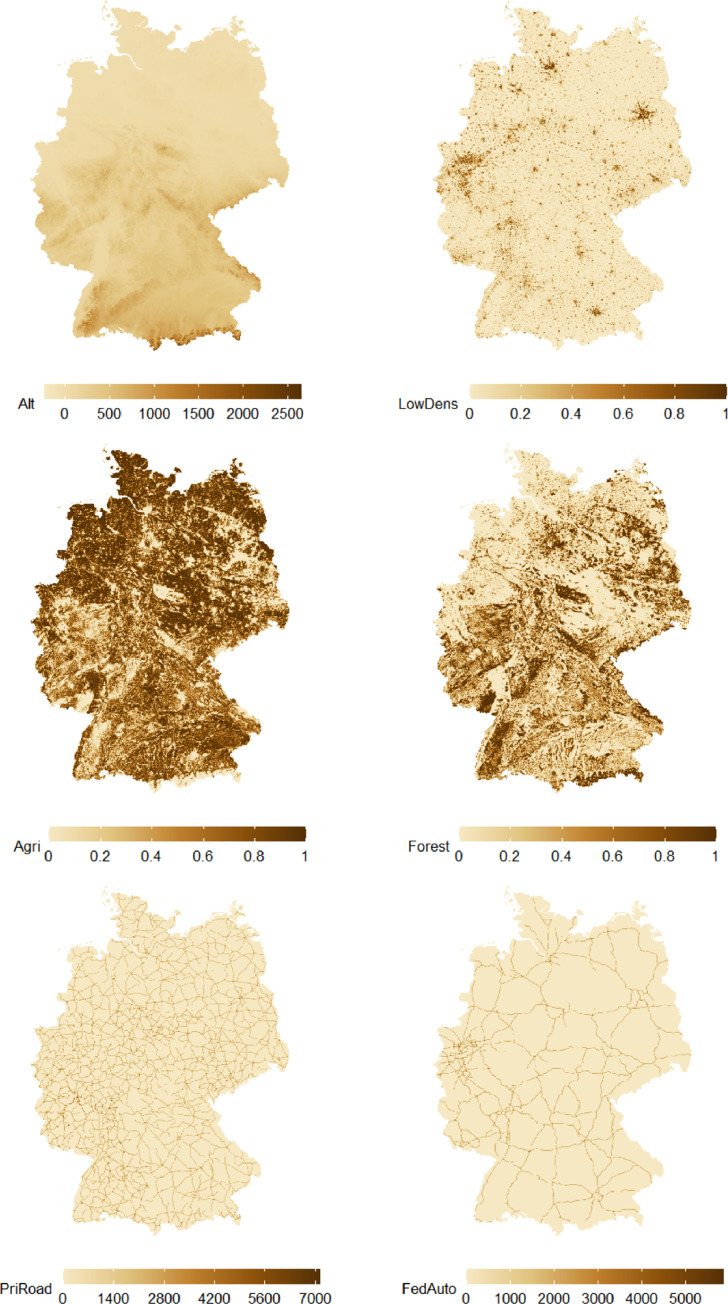
Grid over Germany in 1 × 1 km resolution in dataset gridDE; grid cells colored according to values of variables Alt (top left), LowDens (top right), Agri (middle right), Forest (middle right), PriRoad (bottom left), and FedAuto (bottom right); darker shades of brown indicate higher variable values.

The maps in Fig. 3 illustrate the values of six variables provided in dataset gridDE. Each map is based on the grid over Germany in 1 × 1 km resolution and grid cells are colored according to the values of the respective variable. The top left plot refers to altitude (Alt) and darker shades of brown correspond to grid cell centers located higher above sea level. The top right, middle left, and middle right plot refer to the proportion of land covered by low density residential area (LowDens), agricultural area (Agri), and forestry area (Forest), respectively; values range from zero to one and darker shades of brown correspond to grid cell centers that exhibit a higher percentage of the respective land use in a buffer of radius 1 km. The two plots at the bottom of the figure refer to the length of primary roads (PriRoad; left plot) and federal autobahn (FedAuto; right plot); darker shades of brown correspond to grid cell centers with higher values of road lengths for the respective road type in a buffer of radius 1 km.

Figs. 1–3 were created using R-packages cowplot [10], data.table [11], ggplot2 [12], RColorBrewer [13], and sp [14], [15].

## 2 Experimental Design, Materials and Methods

The following bullet points detail where the raw data are retrieved.

• Mean annual NO_2_ concentration levels (in $\mu {\text{g/m}}^{3}$) across Germany for 2015 are available from the German air quality monitoring network provided by the European Environment Agency [3]. The raw data can be downloaded from EEAData by selecting Germany in Data by country and include detailed information on the monitoring sites.
• CORINE land cover data 2012 Version 18 (CLC12) are provided by the European Environment Agency [4] under CorineData. The raw data include information on land cover over Europe. The data are retrieved in raster format (resolution 100 × 100 m): Each raster cell is assigned one of 44 CORINE land cover classes. The data contain 2,661,003 missing values (4.7%). It is described below how missing values are handled when deriving values of the variables.
• The shapefile of the German boundary can be downloaded from GADM database [5] by selecting version 2.8 and country *Germany*.
• The shapefile of the German administrative regions at municipality key level is available from the Federal Government for Geo-Information and Geodesy [6]. The raw data can be downloaded from GermanAdminRegionsData by selecting VG250-EW Ebenen GK3 Shape. The shapefile contains information on the total number of inhabitants of each municipality.
• The shapefile of the road traffic network over Europe is available from EuroGeographics [7]. The raw data can be downloaded from RoadTrafficNetworkData and contain one folder for Europe as a whole and one separate folder for each country. The shapefile called RoadL of the folder referring to Germany is employed; it provides information on four different types of roads: Federal autobahn, primary roads, secondary roads, and local routes.
• The digital terrain model grid of width 200 m is available from the Federal Government for Geo-Information and Geodesy [8]. The raw data can be downloaded from TerrainModelGrid by selecting DGM200 GK3 GRID-ASCII.

The secondary datasets monSitesDE and gridDE were obtained by (pre-)processing the raw data with the two scripts 00_MonSitesGermany.R and 10_GridGermany.R in the statistical software R [2]. Both, datasets and R-scripts are available from the online repository https://doi.org/10.5281/zenodo.5148684 ([9]). The secondary data were obtained as follows: The spatial data were imported into R via function readOGR(). Function spTransform() was used to transform the coordinate reference systems of spatial objects, where necessary. Both functions are available from package rgdal [17].

The values of the variables provided in dataset monSitesDE only (variables marked with * in Table 1) were derived by filtering the raw data from the European Environment Agency [3].

The grid over Germany of resolution 1 × 1 km was constructed based on the grid topology of the CORINE land cover data [4] and an auxiliary shapefile of the German boundary [5]. The grid topology was used to define an empty rectangular grid that was cropped to the shape of Germany via function mask() from package raster [18].

The values of the variables provided in dataset gridDE only (variables marked with ** in Table 1) resulted directly from the construction of the grid. Values for variable Alt were already included in the meta information on the monitoring sites provided by the European Environment Agency [3]. For the grid cell centers in gridDE, values for Alt were derived from the digital terrain model grid via function extract() from package raster.

The required computations to obtain the values of the remaining variables are identical for datasets monSitesDE and gridDE. In the following, the term *location of interest* refers to the location of a monitoring site or grid cell center.

For the variables indicating land use, the cells of CLC12, whose cell center lay within a buffer of radius 1 km around the location of interest, were extracted via function extract() from package raster. The CORINE land cover classes 1-25 attributed to the extracted cells were then grouped into ten classes according to [16]. Table 3 summarizes the grouping.

**Table 3:** Grouping of CORINE classes 1-25 into 10 classes according to [16].

Grouped class	Description	CLC classes
1	High density residential	1
2	Low density residential	2
3	Industry	3
4	Transport	4
5	Seaports	5
6	Airports	6
7	Construction	7–9
8	Urban Greenery	10–11
9	Agriculture	12–22
10	Forest	23–25

Then, the proportion of surface area of each grouped class in the buffers was computed by dividing the number of cells of each grouped class by the total number of cells extracted. The obtained values were attributed to the predictors listed in Table 1. Note that cells with missing values were included in the computation of the total number of cells extracted.

The shapefile of the German municipalities was used to compute the population density at municipality key level. Therefore, the total number of inhabitants of each municipality – as given in the shapefile – was divided by the area of the respective municipality. The latter was approximated by function gArea() from package rgeos [19]. The municipality in which the location of interest is located, was identified via function over() from package sp [14], [15]. The corresponding values for municipality key AGS and population density PopDens were attributed to the location of interest.

Values for the lengths of the different types of roads PriRoad, SecRoad, FedAuto, and LocRoute were computed based on the road traffic network data. First, functions gBuffer() and gIntersects() from package rgeos were applied and the line segments of all federal autobahn, primary roads, secondary roads, and local routes, which lay within a buffer of radius 1 km around the location of interest, were extracted. Second, the lengths of the respective line segments for all road types were summed up using function SpatialLinesLength() from package sp.

Finally, values for IndRegions were derived from AGS, as this variable already gives the German federal state attributable to the location of interest.

## CRediT authorship contribution statement

**Markus Fritsch:** Methodology, Software, Resources, Data curation, Writing – original draft, Writing – review & editing. **Svenia Behm:** Software, Resources, Visualization, Writing – original draft, Writing – review & editing.

## Declaration of Competing Interest

The authors declare that they have no known competing financial interests or personal relationships which have, or could be perceived to have, influenced the work reported in this article.
